# Temporal trend in quality indicators of diabetes care performance among persons with type 2 diabetes in primary care practice: a serial cross-sectional analytical study, 2013/14 to 2021/22

**DOI:** 10.3389/fendo.2024.1359904

**Published:** 2024-10-23

**Authors:** Ratanaporn Awiphan, Woravut Kowatcharakul, Chidchanok Ruengorn, Kajohnsak Noppakun, Kednapa Thavorn, Surapon Nochaiwong

**Affiliations:** ^1^ Department of Pharmaceutical Care, Faculty of Pharmacy, Chiang Mai University, Chiang Mai, Thailand; ^2^ Pharmacoepidemiology and Statistics Research Center (PESRC), Faculty of Pharmacy, Chiang Mai University, Chiang Mai, Thailand; ^3^ Sansai Hospital, Ministry of Public Health, Chiang Mai, Thailand; ^4^ Division of Nephrology, Department of Internal Medicine, Faculty of Medicine, Chiang Mai University, Chiang Mai, Thailand; ^5^ Ottawa Hospital Research Institute, Ottawa Hospital, Ottawa, ON, Canada; ^6^ Institute of Clinical and Evaluative Sciences (ICES), Ottawa, ON, Canada; ^7^ School of Epidemiology and Public Health, Faculty of Medicine, University of Ottawa, Ottawa, ON, Canada

**Keywords:** quality indicator, patterns, practice care, primary care, time trends, type 2 diabetes

## Abstract

**Background:**

Although the national-based policy implemented an initiative program to offer diabetes care management in Thailand, there are limited time trends of evidence to gauge whether the quality of diabetes care in primary care practice is improving. As such, we aimed to identify temporal trends in the quality of diabetes care performance among type 2 diabetes mellitus (T2DM) patients in primary care practice.

**Methods:**

Using assembled patient-level data from a suburban community in northern Thailand, this serial retrospective cross-sectional analytical study obtained adult T2DM patients from nine consecutive fiscal years 2013/14 (*n* = 976) to 2021/22 (*n* = 1,242). Based on international and national guidelines recommended, nine quality indicators were examined, namely, smoking cessation, hemoglobin A1c monitoring, foot and eye examinations, albuminuria testing, statin prescription, angiotensin-converting enzyme inhibitor/angiotensin II receptor blocker (ACEI/ARB) prescription for chronic kidney disease (CKD)/albuminuria, and blood pressure and glycemic control. Rates and time trends achieved in each quality indicator performance were estimated. Differences in the rates of patients who met each quality indicator across reimbursement schemes were explored.

**Results:**

From 2013/14 to 2021/22, all quality indicators have increased over time (*p* for trend <0.05) except for smoking cessation, which remained steady. In 2021/22, only three out of nine quality indicators (i.e., smoking cessation, annual HbA1c monitoring, and annual foot examination) were successfully met at 70% or greater. Differences in quality indicators of diabetes performance were observed, particularly those under the Civil Servant Medical Benefit Scheme compared with other health insurance counterparts. For overall time trends analysis (compared with 2013/14), significant relative changes in the fiscal year 2021/22 were found in the annual foot examination (adjusted 12.1% increase; *p* = 0.048), annual albuminuria testing (adjusted 12.1% increase; *p* = 0.048), and ACEI/ARB prescription for persons with CKD or albuminuria (adjusted 22.2% increase; *p* = 0.025).

**Conclusion:**

Among adult T2DM patients from 2013/14 to 2021/22, overall quality indicators for diabetes performance have substantially improved over time. However, health inequity regarding diabetes care performance was found across different reimbursement schemes. Sustainable policy implementation and innovative strategies to narrow health inequity are warranted to optimize diabetes care in primary care practice.

## Introduction

According to the Global Burden of Diseases, Injuries, and Risk Factors Study, diabetes is one of the contributing causes of disability and has been recognized as a major cause of noncommunicable disease (NCD) deaths (1, 2). By 2050, it has been projected that more than 1.31 billion people will be living with diabetes worldwide, more than 90% of whom will have type 2 diabetes mellitus (T2DM) (2). People living with T2DM, particularly among those with diabetes-related complications, place immense strains on the disease burden, and they also experience impaired health-related quality of life (3). In addition, it is predicted that T2DM and its consequences consume approximately 12% of worldwide healthcare utilization (4). In Thailand, although the prevalence of diabetes has remained stable at 10% (ranging from 7.7% to 9.9%) over the last two decades (2, 4–7), it is lower in 43 countries and territories worldwide in 2021 (2), and diabetes remains a major public health burden in terms of morbidity, mortality, and healthcare expenditure.

In recent years, collaborative international societies [i.e., American Diabetes Association (ADA), American College of Cardiology/American Heart Association (ACC/AHA), and Kidney Disease: Improving Global Outcomes (KDIGO)] (8–10) and national professional societies (11, 12) have endorsed evidence-based clinical practice guidelines for entities of diabetes care, such as process of diabetes monitoring, optimal target for glycemic and blood pressure (BP) control, and medication optimization, to delivery of effective treatments and services and high-quality diabetes care. However, national and international literature evidence consistently underscores major gaps in delivering health services based on guideline recommendations and implementation in diabetes practice care (13–16).

Over a decade ago, the Ministry of Public Health in Thailand implemented an area health initiative program—Service Plan, which offers diabetes care management as part of the standard of care in NCD healthcare services for integrating and continuing care at primary care practices (11). Since 2013, this national policy framework has been employed at the local and regional levels in an effort to improve the quality of diabetes care, including expanded process of care and quality of measurement, adopted reporting quality of program performance, improved coverage for recommended services and therapy using evidence-based practice, and extension of transition of care across different level of services (11).

Despite these national policy efforts, there are limited time trends of evidence to gauge whether the quality of diabetes care in primary care practice is improving over time. Available studies to date in the Thai diabetes population have been limited by attention to only specific healthcare settings (e.g., elderly T2DM, specialist clinic, or tertiary care) (15, 17, 18) or limited generalizability by focusing on particular source services (5). Regarding studies in primary care settings, most of them have yielded only 1-year or short periods of diabetes care performance figures (16, 19, 20) or reporting before the implementation of the Service Plan—national policy (21), which precludes a temporal appraisal of change and remains a challenge in contemporary diabetes care practices.

To address this knowledge gap in primary care practice in Thailand, we measured nine simple quality indicators that are readily reported and available in primary care practices, involving preventive care, the process of care and testing, and medication utilization and disease control-based outcomes in the clinical entities of national and international guidelines’ recommended care. Based on a serial cross-sectional study between fiscal year 2013/14 and 2021/22 in a primary care setting within a suburban community in northern Thailand, we aimed to investigate whether national-based policy efforts for diabetes care performance using simple quality indicator metrics have been successful over time. Moreover, to address gaps in access to health services and coverage of important diabetic care, we investigated differences in diabetes care quality by health insurance status.

## Materials and methods

### Study design

We conducted a repeated retrospective cross-sectional analytical study from a single-center cohort of T2DM patients from the Sansai Hospital, a suburban community in northern Thailand, between 1 October 2012 and 30 September 2022. Because of the nature of retrospective data collection and the utilization of de-identified information, the requirement for informed consent was waived. We obtained institutional review board approval from the Chiang Mai Provincial Public Health Office (40/2563). We followed the Declaration of Helsinki and the amendments or comparable ethical standards and reported according to the Strengthening the Reporting of Observational Studies in Epidemiology (STROBE) Statement guidelines (22).

### Study population

We conducted a serial cross-sectional study that includes data on T2DM patients in primary care practices from nine consecutive fiscal years (October 1 to September 30), namely, 2013/14, 2014/15, 2015/16, 2016/17, 2017/18, 2018/19, 2019/20, 2020/21, and 2021/22. Within the measurement fiscal year, we included adult patients (≥18 years of age) diagnosed with T2DM and enlisted at least two consecutive outpatients in primary care practice encounters during that fiscal year. We identified patients with T2DM diagnosis within the following fiscal year based on the International Statistical Classification of Diseases, Ninth Revision (ICD-9) and Tenth Revision (ICD-10) Clinical Modification codes. To minimize the missing values and ensure adequate ascertainment of information, we excluded patients who had periods of medical and pharmacy coverage less than 365 days before the index date of the following fiscal years.

### Clinical variables

Variables regarding T2DM patients and diabetes care were based on the information available at primary care practice. These datasets included electronic health record (EHR)—outpatient and inpatient data, administrative data on pharmacy claims, laboratory results, and patient-level data on primary care practice on diabetes and complications. Based on public health protection schemes, we categorized health insurance status into three groups: the Universal Coverage Scheme (UCS) by the National Health Security Office, the Civil Servant Medical Benefit Scheme (CSMBS), and the Social Security Scheme (SSS)/others. We combined those with other health insurance (e.g., self-payment and private insurance) with the SSS group because of the small number of patients, which would preclude the analyses. Smoking status and diabetes care testing [i.e., hemoglobin A1c (HbA1c), foot and eye examination, and urine dipstick/urine albumin–creatinine ratio (UACR)] were extracted from EHR data and primary care practice on diabetes and complications routinely reported data.

Regarding the KDIGO guideline (10), we considered T2DM patients with chronic kidney disease (CKD) using serum creatinine measurements for calculating an estimated glomerular filtration rate (eGFR) based on the Chronic Kidney Disease—Epidemiology Collaboration (CKD-EPI) equation. Those with an eGFR value <60 mL/min/1.73 m^2^ (stage 3–5) for more than 3 months before the index fiscal year were classified as having comorbid CKD. History of comorbid conditions (i.e., hypertension, coronary heart disease, and cerebrovascular disease) was ascertained based on the ICD-9/10 revision.

### Outcomes: quality indicators performance

Regarding the international guidelines and the initiative Service Plan—a program by the Ministry of Public Health of Thailand (8–12), we adapted and employed nine quality indicators for diabetes care performance in the primary care practice based on preventive care (one indicator), process of care and testing (four indicators), and medication use and disease control (four indicators). These quality indicators for diabetes care included (i) smoking cessation (no tobacco use), (ii) annual HbA1c monitoring, (iii) annual diabetic foot examination, (iv) annual eye examination (digital fundus camera), (v) annual urine dipstick test and/or UACR, (vi) diabetes control (HbA1c <8.0%), (vii) BP control (<140/90 mmHg), (viii) statin prescription according to recommended guidelines, and (ix) angiotensin-converting enzyme inhibitors/angiotensin II receptor blockers (ACEIs/ARBs) prescription for persons with CKD or urine albumin excretion >30 mg/24 h (or equivalent). Each quality indicator was captured using merged EHR, pharmacy claims, laboratory results, and patient-level data on primary care practice on diabetes and complications within the measurement fiscal year. Details of each quality indicator and operational definitions for diabetes care outcome performance are described in **Table 1**.

**Table 1:** Quality indicators of care and operational definitions.

Quality Indicators	Care to be Provided to Fulfill an Indicator	Metric Definition
Preventive Care		
Smoking cessation	No tobacco use	% with no tobacco use Numerator: Persons with no tobacco Denominator: Persons with type 2 diabetes
Processes of Care and Testing		
HbA1c measurement	HbA1c measurement at least once yearly	% with HbA_1c_ test Numerator: Persons with a HbA1c assessment Denominator: Persons with type 2 diabetes
Foot examination	Foot examination within 1 year	% with foot examination Numerator: Persons with a foot examination Denominator: Persons with type 2 diabetes
Eye examination	Eye examination within 1 year	% with eye examination Numerator: Persons with an eye examination Denominator: Persons with type 2 diabetes
Urine dipstick test and/or UACR measurement	Urine dipstick test and/or UACR measurement at least once yearly	% with urine dipstick test and/or UACR test Numerator: Persons with a urine dipstick test and/or UACR assessment Denominator: Persons with type 2 diabetes
Drug Use and Disease Control		
Glycemic control	HbA1c <8.0%	% with glycemic control Numerator: Persons with HbA1c level <8.0% Denominator: Persons with assessment of HbA1c with type 2 diabetes
BP control	Systolic BP <140 mmHg and diastolic BP <90 mmHg	% with BP control Numerator: Persons with systolic BP <140 mmHg and diastolic BP <90 mmHg Denominator: Persons with assessment of BP with type 2 diabetes
Statin therapy†	Use of statin therapy according to recommended guidelines: prescription within 1 year	% with use of any statin therapy Numerator: Persons having at least 1 prescription for a statin medication within 1 year of the fiscal years based on recommended guidelines Denominator: Persons with type 2 diabetes (persons with a documented contraindication, intolerance, or allergy to statin therapy automatically meet the statin measure without the requirement for a documented statin prescription)
ACEI or ARB therapy	Use of ACEI or ARBs therapy in persons with CKD or urine albumin excretion >30 mg/24 h (or equivalent)	% with use of ACEIs or ARBs therapy in CKD or albuminuria Numerator: Persons with CKD or albuminuria having at least 1 prescription for an ACEIs or ARB medication within 1 year of the fiscal years Denominator: Persons with type 2 diabetes with albuminuria (persons with a documented contraindication, intolerance, or allergy to ACEI or ARB therapy automatically meet the ACEI or ARB measure without the requirement for a documented ACEI or ARB prescription)

^†^Guideline-recommended statin use is dependent on age, LDL-C level within the past 5 years, and history of cardiovascular disease. Specifically, the following criteria need to be present for the statin indicator to be successfully met: 18 to 20 years of age regardless of LDL-C level (i.e., they are not required to be treated with a statin but meet this metric regardless); 21 to 39 years of age with either an LDL-C level that is less than 190 mg/dL or treatment with a statin; 40 to 75 years of age with either an LDL-C level that is less than 70 mg/dL or treatment with a statin; and any age with a history of vascular disease and either an LDL-C level that is less than 40 mg/dL or treatment with a statin.

ACEIs/ARBs, angiotensin-converting enzyme inhibitors/angiotensin II receptor blockers; BP, blood pressure; CKD, chronic kidney disease; HbA1c, hemoglobin A1c; LDL-C, low-density lipoprotein cholesterol; UACR, urine albumin–creatinine ratio.

### Statistical analysis

Analyses were conducted using Stata version 14.0 (StataCorp LLC, TX). A two-sided test with a *p*-value <0.05 was considered the statistical significance threshold. For descriptive data of patient demographic characteristics, we summarized categorical and continuous variables as the number (percentages) and means ± standard deviations (SDs) or median (min–max).

The unadjusted proportion of T2DM patients who met the diabetes care performance criteria for each quality indicator over the nine consecutive fiscal years (2013/14 to 2021/22) was estimated with 95% confidence intervals (CIs). Using a nonparametric analysis (an extension of the Wilcoxon rank-sum test) for trends across fiscal years, we examined trends in the proportion of patients who met each quality indicator over time. With respect to age- and sex-adjusted analysis, we also estimated diabetes care performance stratified by reimbursement scheme status (i.e., UCS, CSMBS, and SSS/others) to explore inequalities in health benefit package and coverage, which may operate differently among health insurance status. Moreover, the relative change with 95% CIs for each quality indicator performance adjusted for age, sex, and reimbursement scheme was estimated to estimate the temporal trends for change in diabetes care practice between fiscal years 2013/14 and 2021/22.

## Results

### Description of study population

Overall, sociodemographic and clinical characteristics of T2DM were generally similar across study periods (the proportion of female patients ranged from 56.5% to 57.3%, and the proportion with UCS health insurance ranged from 74.6% to 74.9%), with the noticeable difference in mean age increased from 57.9 to 61.4 years. Based on the recent fiscal year (2021/22) data, a total of 1,242 T2DM patients met the inclusion criteria. Characteristics of patients with T2DM in the fiscal year 2021/22 are described in **Table 2** (mean ± SD age, 61.4 ± 10.8 years; 56.5% female; mean body mass index ± SD, 24.7 ± 4.9 kg/m^2^; mean HbA1c ± SD, 8.2 ± 2.1%). Generally, patient characteristics and medication for glycemic and BP control were different between reimbursement schemes (**Table 2**). For example, patients undergoing CSMBS health insurance had a significant difference in a higher proportion of elderly, a higher proportion of long-standing diabetes (>10 years), and a higher level of low-density lipoprotein cholesterol than the other health insurance counterparts (**Table 2**).

**Table 2:** Demographic characteristics of persons with type 2 diabetes according to sex, fiscal year 2021/22.

Characteristic	No. (%)^†^				
	Total (*n* = 1,242)	UCS by NHSO (*n* = 927)	CSMBS (*n* = 160)	SSS/Others (*n* = 155)	*p*-Value
Age, years; mean ± SD	61.4 ± 10.8	61.4 ± 10.6	64.5 ± 9.7	58.5 ± 12.0	0.030
≤55	324 (26.1)	245 (26.4)	22 (13.8)	57 (36.8)	<0.001
56–65	515 (41.5)	383 (41.3)	73 (45.6)	59 (38.1)	
66–75	278 (22.4)	210 (22.7)	39 (24.4)	29 (18.7)	
>75	125 (10.0)	89 (9.6)	26 (16.2)	10 (6.4)	
Sex					
Male	540 (43.5)	410 (44.2)	69 (43.1)	61 (39.4)	0.527
Female	702 (56.5)	517 (55.8)	91 (56.9)	94 (60.6)	
BMI, kg/m^2^; mean ± SD	24.7 ± 4.9	24.7 ± 5.0	24.8 ± 4.0	24.6 ± 5.0	0.004
<18.5	89 (7.4)	69 (7.7)	3 (1.9)	17 (11.3)	0.028
18.5–22.9	367 (30.4)	269 (30.0)	57 (35.8)	41 (27.3)	
23.0–24.9	219 (18.2)	168 (18.8)	27 (17.0)	24 (16.0)	
≥25	530 (44.0)	390 (43.5)	72 (45.3)	68 (45.3)	
Comorbid conditions					
Hypertension	1001 (80.6)	759 (81.9)	129 (80.6)	113 (72.9)	0.036
Coronary heart disease	104 (8.4)	89 (9.6)	7 (4.4)	8 (5.2)	0.026
Cerebrovascular disease	92 (7.4)	76 (8.2)	6 (3.8)	10 (6.4)	0.117
Chronic kidney disease: stage 3–5	313 (25.2)	245 (26.4)	37 (23.1)	31 (20.0)	0.197
Long-standing diabetes (>10 years)	381 (30.7)	291 (31.4)	63 (39.4)	27 (17.4)	<0.001
Systolic BP, mmHg; mean ± SD	131.2 ± 18.5	131.3 ± 18.8	133.1 ± 18.8	128.9 ± 16.2	0.070
Diastolic BP, mmHg; mean ± SD	77.2 ± 11.2	77.2 ± 11.3	76.5 ± 10.6	77.4 ± 11.0	0.544
eGFR mL/min per 1.73 m^2^; mean ± SD	78.4 ± 26.2	77.6 ± 26.6	77.8 ± 22.9	83.4 ± 27.0	0.050
HbA1c, %; mean ± SD	8.2 ± 2.1	8.2 ± 2.1	7.7 ± 1.6	8.0 ± 2.3	<0.001
LDL-C, mg/dL; mean ± SD	115.9 ± 42.1	115.5 ± 42.7	122.9 ± 43.8	111.0 ± 35.6	0.022
Medication for glycemic control					
Diet only	137 (11.0)	91 (9.8)	27 (16.9)	19 (12.3)	0.008
Oral antidiabetic drugs	842 (67.8)	618 (66.7)	111 (69.4)	113 (72.0)	
Oral antidiabetic drugs and insulin	162 (13.1)	135 (14.6)	15 (9.4)	12 (7.7)	
Insulin only	101 (8.1)	83 (9.0)	7 (4.4)	11 (7.1)	
No. of antihypertensive therapy, median (min–max)	1 (0–4)	1 (0–4)	1 (0–3)	1 (0–3)	0.033

^†^Missing data for BMI, 37 (3.0%); HbA1c, 141 (11.4%); LDL-C, 48 (3.9%).

BMI, body mass index; BP, blood pressure; CSMBS, Civil Servant Medical Benefit Scheme; eGFR, estimated glomerular filtration rate; HbA1c, hemoglobin A1c; LDL-C, low-density lipoprotein cholesterol; NHSO, National Health Security Office; SD, standard deviation; SSS, Social Security Scheme; UCS, Universal Coverage Scheme.

### Diabetes care performance according to quality indicators

Temporal trends in the unadjusted proportion of T2DM patients who met each quality indicator of diabetes care performance are illustrated in **Table 3**. All quality indicators have been increased over time except for the domain of prevention care—smoking cessation (*p* for trend <0.05; **Table 3**). Overall, only three out of nine quality indicators (i.e., smoking cessation, annual HbA1c monitoring, and annual foot examination) successfully met 70% or greater in the fiscal year 2021/22.

**Table 3:** Quality indicators of diabetes care performance within the measurement fiscal year.

Measure of Quality Indicators	Percentage of Type 2 Diabetes Patients Who Met the Quality Indicators Performance (95% CI)									
	Fiscal Year 2013/14 (*n* = 976)	Fiscal Year 2014/15 (*n* = 994)	Fiscal Year 2015/16 (*n* = 1,089)	Fiscal Year 2016/17 (*n* = 1,156)	Fiscal Year 2017/18 (*n* = 1,174)	Fiscal Year 2018/19 (*n* = 1,190)	Fiscal Year 2019/20 (*n* = 1,220)	Fiscal Year 2020/21 (*n* = 1,204)	Fiscal Year 2021/22 (*n* = 1,242)	*p*-Value for Trend
Preventive Care										
• Smoking cessation: no tobacco use	90.7 (88.7–92.3)	90.8 (88.9–92.5)	91.2 (89.3–92.7)	91.1 (89.3–92.6)	91.1 (89.3–92.6)	91.1 (89.3–92.6)	91.0 (89.2–92.4)	91.5 (89.8–93.0)	91.5 (89.8–93.0)	0.443
Processes of Care and Testing										
• HbA1c measurement: at least once yearly	79.7 (77.1–82.1)	80.1 (77.5–82.4)	80.5 (78.1–82.8)	83.3 (81.0–85.3)	83.6 (81.3–85.6)	83.8 (81.6–85.8)	83.0 (80.8–85.0)	87.0 (85.0–88.8)	88.6 (86.8–90.3)	<0.001
• Foot examination: within 1 year	68.5 (65.6–71.4)	69.1 (66.2–71.9)	69.0 (66.1–71.6)	71.9 (69.2–74.4)	72.2 (69.6–74.7)	72.6 (70.0–75.1)	70.7 (68.1–73.2)	78.0 (75.6–80.2)	80.5 (78.2–82.6)	<0.001
• Eye examination: within 1 year	59.7 (56.6–62.8)	60.4 (57.4–63.4)	60.0 (57.0–62.8)	63.2 (60.4–66.0)	63.7 (60.9–66.4)	64.1 (61.3–66.8)	62.7 (60.0–65.4)	66.4 (63.6–69.0)	69.2 (66.5–71.7)	<0.001
• Urine dipstick test and/or UACR measurement: at least once yearly	54.6 (51.4–57.7)	54.9 (51.8–58.0)	54.8 (51.8–57.8)	57.5 (54.6–60.3)	57.9 (55.1–60.7)	58.1 (55.2–60.8)	61.6 (58.8–64.2)	64.9 (62.1–67.5)	66.8 (64.2–69.4)	<0.001
Drug Use and Disease Control										
• Glycemic control: HbA1c <8.0%	52.1 (48.8–55.5)	52.3 (49.0–55.6)	52.8 (50.0–55.9)	54.4 (51.3–57.4)	54.5 (51.4–57.5)	54.8 (51.8–57.8)	54.9 (51.9–57.8)	55.5 (52.5–58.4)	55.8 (52.9–58.8)	0.022
• BP control: systolic BP <140 mmHg and diastolic BP <90 mmHg	57.7 (54.6–60.8)	58.1 (55.1–61.2)	58.0 (55.1–60.9)	59.9 (57.1–62.7)	59.9 (57.0–62.6)	59.7 (56.9–62.5)	59.8 (57.0–62.5)	61.3 (58.5–64.0)	61.6 (58.8–64.3)	0.011
• Statin therapy: prescription within 1 year based on recommended guidelines	52.7 (50.0–55.8)	52.9 (49.8–56.0)	54.0 (51.0–56.9)	55.9 (53.0–58.7)	56.4 (53.5–59.2)	56.6 (53.8–59.4)	56.8 (54.0–59.6)	60.0 (57.2–62.7)	59.6 (56.8–62.3)	<0.001
• ACEI or ARB therapy: for persons with CKD or urine albumin excretion >30 mg/24 h (or equivalent)	38.7 (34.4–43.3)	38.5 (34.1–43.0)	40.0 (35.7–44.4)	43.5 (39.3–47.9)	47.7 (43.5–52.0)	54.0 (49.7–58.1)	56.1 (51.9–60.2)	61.2 (57.1–65.2)	60.9 (56.8–64.8)	<0.001

ACEIs/ARBs, angiotensin-converting enzyme inhibitors/angiotensin II receptor blockers; BP, blood pressure; CI, confidence interval; CKD, chronic kidney disease; HbA1c, hemoglobin A1c; UACR, urine albumin–creatinine ratio.

For smoking cessation criteria (**Table 3**), consistent time trends of T2DM patients with no tobacco use were found, ranging from 90.7% (95% CI, 88.7–92.3) in 2013/14 to 91.5% (95% CI, 89.8–93.0). For the domain of process of care and testing, the proportion of T2DM patients who met diabetes care performance was highest among the criteria of annual HbA1c monitoring (ranging from 79.7% to 88.6%) and lowest among the criteria of annual urine dipstick test and/or UACR measurement (ranging from 54.6% to 66.8%). Of these, during the fiscal year 2019/20, there was a slight drop in the prevalence rate of annual HbA1c monitoring and annual foot and eye examinations.

Likewise, there was an increase in medication use and disease control during the fiscal year 2013/14 to 2021/22: 52.1% to 55.8% for the criteria of HbA1c <8.0%; 57.7% to 61.6% for the criteria of systolic and diastolic BP <140/90 mmHg, and 52.7% to 59.6% for the use of statin therapy based on recommended guidelines (**Table 3**). Interestingly, the use of ACEI/ARBs therapy for persons with CKD or urine albumin excretion >30 mg/24 h (or equivalent) had dramatically increased from 38.7% (95% CI, 34.4–43.3) in 2013/14 to 60.9% (95% CI, 56.8–64.8) in 2021/22.

**Figure 1:**
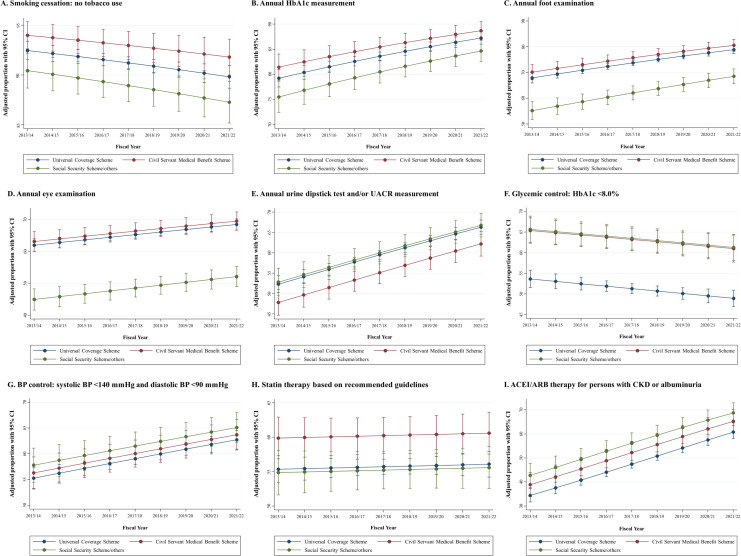
**(A–I)** Age-, sex-, and reimbursement-adjusted of trends quality indicators of diabetes care performance between the fiscal year 2013/14 and 2021/22. ACEI/ARB, angiotensin-converting enzyme inhibitors/angiotensin II receptor blockers; BP, blood pressure; CI, confidence interval; CKD, chronic kidney disease; HbA1c, hemoglobin A1c.

After adjusting for age and sex, T2DM patients with CSMBS health insurance revealed time trends in a greater proportion of patients who met each quality indicator of diabetes care performance compared with UCS or SSS/other groups, particularly for foot and eye examination, glycemic control, and statin therapy (**Figure 1**). Compared with the inception of study period (2013/14; **Table 4**), significant relative change in the fiscal year 2021/22 were found in the quality indicator of annual foot examination (adjusted 12.1% increase; 95% CI, 0.2% to 24.0%; *p* = 0.048), annual urine dipstick test and/or UACR measurement (adjusted 12.1% increase; 95% CI, 1.7% to 24.0%; *p* = 0.048), and use of ACEI/ARB therapy for persons with CKD or urine albumin excretion >30 mg/24 h (or equivalent) (adjusted 22.2% increase; 95% CI, 4.5% to 39.9%; *p* = 0.025).

**Table 4:** Trends in quality indicators of diabetes care between fiscal year 2013/14 and 2021/22.

Measure of Quality Indicators	Percent of Type 2 Diabetes Who Met the Quality Indicators Performance (95% CI)		% Unadjusted Relative Change (95% CI)	*p*-Value	% Adjusted Relative Change (95% CI)^†^	*p*-Value
	Fiscal Year 2013/14 (*n* = 976)	Fiscal Year 2021/22 (*n* = 1,242)				
Preventive Care						
• Smoking cessation: no tobacco use	90.7 (88.7–92.3)	91.5 (89.8–93.0)	0.8 (−9.6 to 11.3)	0.849	0.9 (−11.0 to 12.8)	0.841
Processes of Care and Testing						
• HbA1c measurement: at least once yearly	79.7 (77.1–82.1)	88.6 (86.8–90.3)	8.9 (−1.5 to 19.4)	0.082	8.9 (−3.0 to 20.8)	0.106
• Foot examination: within 1 year	68.5 (65.6–71.4)	80.5 (78.2–82.6)	12.0 (1.5 to 22.4)	0.031	12.1 (0.2 to 24.0)	0.048
• Eye examination: within 1 year	59.7 (56.6–62.8)	69.2 (66.5–71.7)	9.2 (−2.2 to 20.7)	0.096	9.0 (−5.6 to 23.5)	0.162
• Urine dipstick test and/or UACR measurement: at least once yearly	54.6 (51.4–57.7)	66.8 (64.2–69.4)	12.2 (1.8 to 22.7)	0.029	12.1 (1.7 to 24.0)	0.048
Drug Use and Disease Control						
• Glycemic control: HbA1c <8.0%	52.1 (48.8–55.5)	55.8 (52.9–58.8)	1.6 (−17.6 to 20.9)	0.844	3.4 (−19.7 to 26.4)	0.704
• BP control: systolic BP <140 mmHg and diastolic BP <90 mmHg	57.7 (54.6–60.8)	61.6 (58.8–64.3)	3.9 (−6.5 to 14.4)	0.396	3.6 (−8.3 to 15.5)	0.449
• Statin therapy: prescription within 1 year based on recommended guidelines	52.7 (50.0–55.8)	59.6 (56.8–62.3)	6.9 (−3.6 to 17.4)	0.157	7.1 (−4.8 to 19.0)	0.175
• ACEI or ARB therapy: for persons with CKD or urine albumin excretion >30 mg/24 h (or equivalent)	38.7 (34.4–43.3)	60.9 (56.8–64.8)	22.2 (6.8 to 37.6)	0.012	22.2 (4.5 to 39.9)	0.025

^†^Adjusted for age, sex, and reimbursement scheme.

ACEIs/ARBs, angiotensin-converting enzyme inhibitors/angiotensin II receptor blockers; BP, blood pressure; CI, confidence interval; CKD, chronic kidney disease; HbA1c, hemoglobin A1c; UACR, urine albumin–creatinine ratio.

## Discussion

In this serial cross-section study of Thai individuals with T2DM, we found an increase in the quality indicator metrics over the nine consecutive fiscal years (2013/14 to 2021/22), reflecting a health system response to the Service Plan, national-based policy efforts for diabetes care management at primary care practices. However, we identified the disparities and gaps in health insurance status for diabetes care quality-based reimbursement in relation to diabetes monitoring and testing, as well as medication utilization based on recommended guidelines and disease control.

Regarding our findings, we underscore a need to understand reasons and identify factors associated with appropriate or poor performance in each diabetes quality indicator. Based on the nine quality indicators metrics measured, we found only three indicators that illustrated satisfactory performance, with criteria fulfilled at 70% or over in recent years. Specifically, the three indicators that illustrated satisfactory performance were in the domains of preventive care (i.e., smoking cessation: no tobacco use) and the process of care and testing (i.e., annual HbA1c monitoring and annual foot examination). However, inadequate quality indicator metrics performance was concerned within the domains of processes of care and testing (i.e., annual eye examination and urine dipstick test and/or UACR measurement at least once yearly) and medication use and disease control [i.e., HbA1c <8.0%, systolic and diastolic BP <140/90 mmHg, use of statin therapy based on recommended guidelines, and use of ACEI/ARBs therapy for persons with CKD or urine albumin excretion >30 mg/24 h (or equivalent)]. In this circumstance, there are substantial opportunities to improve the quality of diabetes care performance, particularly in the domains of processes of care and testing, medication optimization, and disease control.

Collectively, we found that the quality indicators of diabetes care performance among adult Thai T2DM patients have improved over time compared to the previous studies in the past decade (15, 16, 18, 19, 21, 23). Not surprisingly, during the early COVID-19 pandemic, March 2020 in Thailand, the performance rates of quality indicators requiring in-center testing (i.e., HbA1c measurement, foot examination, and eye examination) appeared to be slightly decreased in the fiscal year 2019/20 compared to the previous fiscal year. However, after that, the overall quality indicator metrics rebounded and continued to increase in 2020/21 and 2021/22. We postulate that rapid responses and social community buffer through hospital preparedness with primary care units and village health volunteers may help mitigate the impact of this unprecedented public health event.

In our study, after adjusting for age, sex, and reimbursement scheme, substantial relative changes in quality indicators over the periods of policy implemented were found in the rate of annual foot examination (12.1% increase), annual urine dipstick test and/or UACR measurement (12.1% increase), and a prescription filled for ACEI/ARB therapy among those with CKD or albuminuria (22.2% increase). Although the performance of the process of care delivery and measurement testing has improved over nearly a decade, lower achievement of disease outcome-based performance in terms of BP and glycemic control (<140/90 mmHg and HbA1c <8.0%) remains observed. Notably, these suggested that only attention to the process of care delivery may be inadequate in isolation regarding the burden of diabetes care. Ultimately, upstream interventions that address both individuals (e.g., modifiable risk factors or social determinants of health) and healthcare system levels (e.g., health information systems, innovative health technologies, and integrative care services and resource delivery) may help further mitigate to achieve high-quality diabetes care.

More importantly, despite the fact that Thailand has implemented UCS health insurance since 2002 and covered over 99% of the population, the unmet attrition to the diabetes care continuum was observed (total unmet need was 70%) among persons under the UCS health insurance in recent years (5). Our findings expanded to the previous study by identifying the disparities and gaps in the quality indicators of diabetes care performance among the different reimbursement schemes. Apparently, we found that those under the CSMBS health insurance had fulfilled and met each quality indicator metric more than UCS or SSS/other counterparts. Of these, persons with CSMBS health insurance were more likely to have annual diabetes foot and eye examination, more likely to have glycemic control with HbA1c <8.0%, and more likely to have filled a prescription for statin therapy than persons with UCS or SSS/other health insurance counterparts. However, the reasons for the inequality in access to the standard of diabetes care are unclear and not fully elucidated by sex and age in this study. Unmeasured or residual confounders regarding multifactorial clinical characteristics and healthcare system environment remain a possible explanation. Based on different health benefit packages in healthcare coverage, we postulated that CSMBS health insurance populations may have access to high-quality diabetes care in terms of measurement testing, monitoring, medication optimization, or referral and collaborative care with a specialist professional (i.e., endocrinologist or nephrologist) compared to UCS or SSS/other health insurance counterparts. To improve diabetes care delivery across all health insurance, strategies or innovative policy-based interventions are needed to narrow health inequity across multiple reimbursement schemes.

### Strengths and limitations

As part of a continuous quality improvement program, our findings were based on routinely available serial data collection, reflecting the primary care practice service patterns on diabetes care performance. We highlight the quality indicators of the process of care, health services, and outcome-based performance under the area health initiative strategy that implemented comprehensive diabetes care in primary care centers.

Nevertheless, our study has several limitations. Firstly, this study was based on a single center and unique practice of the Sansai Hospital, which integrated services delivery and coordinated with primary care units and village health volunteers for continuity of care in the community. Thus, the generalizability of these findings to settings other than primary care should be interpreted with caution. However, to the best of our knowledge, we utilize longitudinal data to assess the quality of diabetes care in primary care practice, and some of it may shed light on Thai health policies over time. Secondly, on the basis of our study design, we could not establish whether changes in diabetes care treatment, particularly for medication optimization, were reflected in changes in individuals’ practice of prescribers or due to a policy driven by the Ministry of Public Health. Moreover, we also lacked information regarding the specialties of prescribers (i.e., general physician, endocrinologist, or nephrologist), which may differ in prescribing practice and behavior. Thirdly, based on measurements available in primary care, we limited comorbid CKD to only T2DM patients with moderate to advanced stages (stages 3–5 of CKD) because quantifying eGFR using serum creatinine-based methods cannot identify patients with early stages of CKD. Moreover, urinary protein tests for UACR monitoring were not routinely available in our primary care practice, especially in the early phase of the Service Plan—policy implemented, resulting in the rate of use of ACEI/ARB therapy in persons with CKD being underestimated. Fourthly, on the basis of a temporal trends analysis, medication use for T2DM patients may not be fully elucidated in some clinical situations, for instance, withholding of ACEI/ARB in the clinical entities of hypotension, hyperkalemia, or acute kidney injury. Moreover, we do not include the new treatments, such as glucagon-like peptide-1 receptor agonists or sodium-glucose cotransporter-2 inhibitors, which reduce the risk of CKD progression and mortality among persons with T2DM (8). As such, these promising drugs need to be prioritized in this area for further studies regarding the quality of diabetes in primary care practices. Finally, although we found inequalities in terms of health insurance status, an individual’s level of social determinants of health gradients (e.g., socioeconomic position, education, working life conditions, food insecurity, and access to affordable health services of decent quality) was not fully investigated in this study, which may explain the differences in the proportion of success in each quality indicator of diabetes care performance over time. In this regard, further studies should pay attention to the influence of non-medical factors such as social health inequalities and access to effective diabetes care services.

### Conclusions and policy implications

We found that the overall diabetes care performance among adult T2DM patients has substantially improved over time for preventive care, process of care and testing, and medication use and disease control in a primary care practice from the fiscal years 2013/14 to 2021/22. Based on our findings, the relative change in diabetes care practice patterns reflected a shift in the continuous quality improvement system and the impact of the area health initiative program offering diabetes care at primary care centers. However, inequalities in health insurance status were observed; compared with UCS or SSS/other counterparts, T2DM patients undergoing the CSMBS health benefits package were more likely to have foot and eye examinations, achieve glycemic control, and have statin therapy.

Given prominent health insurance status disparities, we underscore that the unmet need for collaborative care remains a challenge for improving quality indicators of diabetes care and minimizing inequality in access to the standard healthcare system. We propose that proactive interprofessional collaborative teams and long-term holistic care services will bring highly effective coverage care to improve diabetes care performance, particularly for diabetes control and medication optimization. Moreover, the sustainable policy that implemented and incorporated innovative approaches driven by evidence-based decisions will be necessary to optimize diabetes care in primary care practice.

## Data Availability

The raw data supporting the conclusions of this article will be made available by the authors, without undue reservation.
